# STING agonist protects against exacerbation of schistosome egg-induced immunopathology

**DOI:** 10.1371/journal.ppat.1014394

**Published:** 2026-07-10

**Authors:** Pengyu Liu, Megan S. Linnane, Kaile Jump, Shuchang Tian, Santoshi Chaudhary, Rajeswaran Mani, Jordan E. Bisanz, Parisa Kalantari

**Affiliations:** 1 Department of Veterinary and Biomedical Sciences, Center for Molecular Immunology and Infectious Disease, Pennsylvania State University, University Park, Pennsylvania, United States of America; 2 Department of Biochemistry and Molecular Biology, Pennsylvania State University, University Park, Pennsylvania, United States of America; 3 Huck Institutes of Life Sciences, Pennsylvania State University, University Park, Pennsylvania, United States of America; Rush University Medical Center, UNITED STATES OF AMERICA

## Abstract

Schistosomiasis, caused by parasitic worms, affects over 250 million people globally, with limited treatment options due to praziquantel’s inability to prevent reinfection or reduce immunopathology. In *Schistosoma mansoni* (*S. mansoni*) infection, egg-induced granulomatous inflammation in the liver and intestines is driven by CD4 T helper (Th) cell responses, with severe pathology in some individuals mediated by Th17 cell activation. We previously demonstrated that the stimulator of interferon genes (STING) mitigates schistosome egg-induced immunopathology by promoting type I Interferon (IFN-I) production and suppressing Th17 responses. Here, we investigate the therapeutic potential of the STING agonist diABZI-3 in a high-pathology CBA mouse model. In vitro, diABZI-3 pretreatment of bone marrow-derived dendritic cells (BMDCs) significantly enhanced IFNβ production while abolishing IL-1β and IL-17 expression in response to schistosome egg stimulation, an effect dependent on early administration. In vivo, a single dose of diABZI-3 administered to *S. mansoni*-infected CBA mice reduced liver and intestine granuloma size, lowered IL-1β, IL-17, and CD209a levels, promoted the Foxp3⁺ regulatory T cells, reduced Th17 recruitment, and mitigated inflammation-associated shifts in gut microbiota populations. Furthermore, blocking STING degradation with bafilomycin A1 sustained STING signaling, leading to pronounced IL-1β suppression. These findings highlight diABZI-3 as a promising therapeutic agent for reducing schistosome-induced immunopathology.

## Author summary

Schistosomiasis, caused by the blood fluke genus Schistosoma, is the second most prevalent parasitic disease worldwide, infecting over 250 million people. The disease’s pathology is derived from CD4 + T cell-mediated hepato-intestinal granulomatous inflammatory response to the parasite eggs. Severe symptoms, including liver fibrosis, portal hypertension, splenomegaly, gastrointestinal hemorrhage, and death, occur in 5–10% of cases. Currently, praziquantel, an anthelmintic, is the only treatment available, a drug that is only effective against adult worms. This study proposes a novel therapeutic approach to reduce disease severity by targeting STING. Our results demonstrate that the STING agonist decreases proinflammatory cytokine production in response to schistosome egg stimulation. Furthermore, STING agonist mitigates pathology in a high-pathology mouse model of schistosomiasis, consistent with an anti-inflammatory liver and intestine cellular environment and gut microbiome composition. Mechanistically, the STING agonist induces STING degradation; however, inhibiting V-ATPase activity blocks STING degradation and enhances IL-1β suppression. These findings suggest a novel therapeutic strategy to complement antiparasitic treatment and mitigate disease-associated organ damage.

## Introduction

Schistosomiasis infects over 250 million people worldwide and causes 3.3 million disability-adjusted life years (DALYs) each year [1]. Currently, the only available treatment for this disease is praziquantel, which is inadequate due to failure to significantly reduce reinfection and disease transmission [2]. Despite extensive vaccine research spanning decades, which has involved identifying and evaluating numerous candidate antigens, none have demonstrated consistently high and acceptable levels of protection. Thus, the development of new therapeutic approaches is increasingly important in the face of drug resistance and vaccine failure [3].

In the case of the species *Schistosoma mansoni (S. mansoni)*, the resulting granulomatous inflammation and fibrosis takes place in the liver and intestines, where trapped parasite eggs trigger a CD4 T helper (Th) cell–mediated granulomatous inflammation and fibrosis. The severity of disease varies significantly from individual to individual but in a minority of patients, there is severe disease and death. *S. mansoni* infection in a murine model similarly leads to dramatic strain variation of immunopathology. In C57BL/6 (BL/6) strain, there is relatively mild hepatic pathology arising in a Th2-dominated cytokine environment [4]. In contrast, CBA mice develop more severe lesions mostly mediated by proinflammatory IL-17-producing Th17 cells [5,6]. Our previous studies highlight a decisive role for the stimulator of interferon genes (STING) in shaping subsequent T cell skewing and immunopathology, as schistosome-infected STING deficient mice display markedly enhanced Th17 cell responses and hepatic granulomatous lesions [7]. Additionally, the pore-forming protein gasdermin D (Gsdmd) was highly expressed in CBA DCs, leading to reduced expression of cGAS/STING, diminished IFNβ, and heightened pyroptosis, when stimulated with eggs [7].

STING is widely recognized for its role in inducing type I Interferon (IFN-I) production. STING resides on the endoplasmic reticulum (ER) and upon activation, translocates to the Golgi apparatus, where it phosphorylates TANK-binding kinase 1 (TBK1) and interferon regulatory factor 3 (IRF3).

Previous reports indicate that STING degradation via trafficking requires its exit from the ER and sorting of STING vesicles to lysosomes for degradation [8]. A key limitation of STING agonists as therapeutics is their typically transient signaling activation, which restricts their efficacy. Elucidating the molecular mechanisms underlying STING degradation is crucial, as inhibiting this pathway can enhance STING activation and IFN-I production, thereby reducing inflammation in schistosomiasis and various other diseases.

The gut microbiome is a potent modulator of host immune responses [9,10] and to infections like schistosomiasis [11–13]. Further, STING deficiency is associated with gut microbiota dysbiosis which may result in a loss of colonization resistance to enteric pathogens [14]. STING is also capable of directly sensing cyclic dinucleotides produced by gut microbes as signaling molecules. Taken together, these demonstrate a need to understand how gut microbes a mediator of disease outcome and an opportunity may be to serve as a biomarker of disease activity [15].

Our previous study demonstrated a protective role of STING in suppressing egg-induced immunopathology. Furthermore, BL/6 dendritic cells expressed higher levels of STING compared to those from CBA mice, resulting in increased levels of IFN-I [7]. Diamidobenzimidazole-based compounds such as diABZI-3 are small molecule STING agonists which have superiority compared to other cyclic dinucleotide STING agonists in terms of potency, tissue penetrance, and specificity [16,17]. Here, we found that diABZI-3 suppresses IL-1β expression in DCs and protects against severe schistosomiasis in vivo. We also found that blocking STING degradation led to more efficient IL-1β degradation, suggesting that this may serve as a more superior therapeutic strategy. We also revealed that infection drove a distinct fecal microbiome profile which could be partly rescued through administration of diABZI3 into mice characterized by altered abundances of *Eggerthellaceae* and *Alistipes* spp., clades associated with modulation of Th17 populations and protective effects against inflammatory diseases such as colitis [18–20].

## Results

### STING agonist, diABZI-3, induces IFN-I production and suppresses IL-1β and IL-17 production in BMDCs

**Fig 1:**
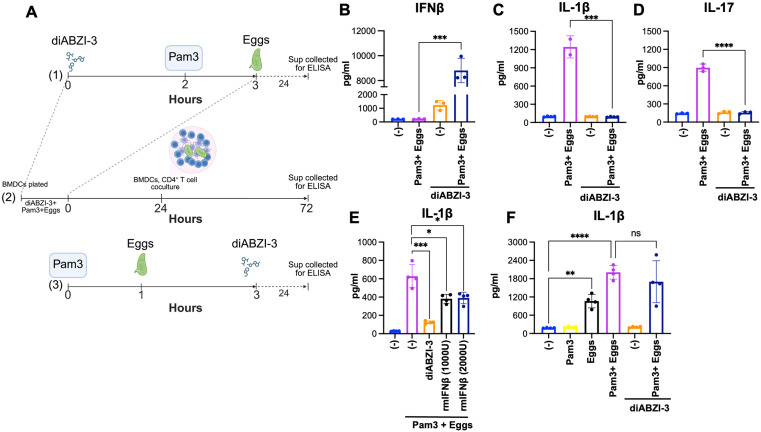
STING agonist diABZI-3 induces IFNβ production and impairs IL-1β and IL-17 production. **(A)** Schematic illustration of BMDCs treated with (1) diABZI-3, Pam3, eggs (2) diABZI-3, Pam3, eggs + co-culture with T cells, (3) Pam3, eggs, diABZI-3 (created in BioRender. Liu, **P.** (2026) https://nam10.safelinks.protection.outlook.com/?url= https%3A%2F%2Fbiorender.com%2Fwnmjqkl&data=05%7C02%7Cpvl5229%40psu.edu%7Ca555926cfa114b54629008decad476fa%7C7cf48d453ddb4389a9c1c115526eb52e%7C0%7C0%7C639171211031269095%7CUnknown%7CTWFpbGZsb3d8eyJFbXB0eU1hcGkiOnRydWUsIlYiOiIwLjAuMDAwMCIsIlAiOiJXaW4zMiIsIkFOIjoiTWFpbCIsIldUIjoyfQ%3D%3D%7C0%7C%7C%7C&sdata=w0BiN4SSNhijSg0%2BnaSoNwCGp5w6lGV5k6I7GEKISW0%3D&reserved=0). **(B-C)** BMDCs were pretreated with diABZI-3 (0.1µM) for 2h followed by stimulation with Pam 3 (1µg/ml) for 1h and then 100 eggs for 24h (schematic (1)). **(D)** Stimulated BMDCs were treated with diABZI-3, Pam3, and eggs as described in (1) followed by co-culture with CD4^+^ naive T cells for 72h. **(E)** Cells were pretreated with diABZI-3 or indicated concentrations of recombinant murine IFNβ (rmIFNβ) for 2h followed by stimulation with Pam3 for 1h and then eggs for 24h. **(F)** BMDCs were treated with Pam3 for 1h followed by stimulation with 100 eggs for 2 h, followed by diABZI-3 (0.1µM) treatment for 24h (schematic (3)). All cytokines (IFNβ, IL-1β and IL-17) in the supernatants in B, C, D, E and F were measured by ELISA. All BMDCs in this Figure and all experiments throughout this manuscript are from CBA mice unless mentioned otherwise. Bars represent the mean + /- SD cytokine levels of three to four biological replicates from one representative experiment of two with similar results. For this and all figures: * p ≤ 0.05, ** p ≤ 0.005, *** p ≤ 0.0005, **** p ≤ 0.00005; ns, not significant.

**Fig 2:**
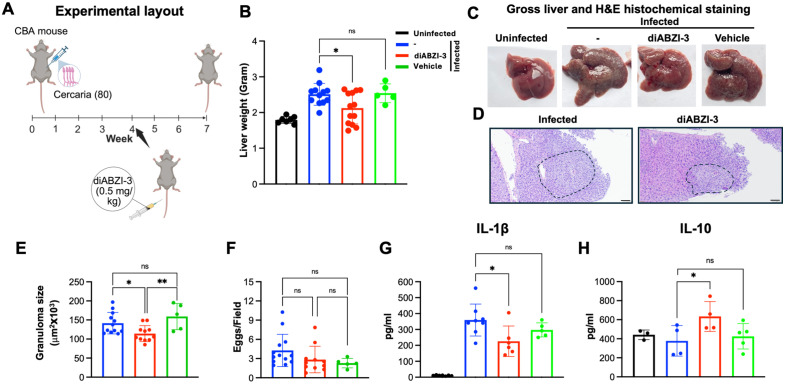
diABZI-3 administration results in reduced egg-induced granulomatous inflammation. **(A)** Schematic illustration of mouse infection with *S. mansoni*. CBA mice infected with S. mansoni and treated with respective treatments [control uninfected (-), Infected (-), infected mice injected with a single dose of 0.5mg/kg of diABZI-3 at 4 weeks post-infection, and infected mice treated with vehicle control (LAL water)] were sacrificed at 7 weeks post-infection (created in BioRender. Liu, P. (2026) https://nam10.safelinks.protection.outlook.com/?url=https%3A%2F%2Fbiorender.com%2Fsf5lvtp&data=05%7C02%7Cpvl5229%40psu.edu%7Ca555926cfa114b54629008decad476fa%7C7cf48d453ddb4389a9c1c115526eb52e%7C0%7C0%7C639171211031286768%7CUnknown%7CTWFpbGZsb3d8eyJFbXB0eU1hcGkiOnRydWUsIlYiOiIwLjAuMDAwMCIsIlAiOiJXaW4zMiIsIkFOIjoiTWFpbCIsIldUIjoyfQ%3D%3D%7C0%7C%7C%7C&sdata=QR1N9%2Bl574OoKdHafF4sjrKfU%2BlyIMoVpOEqQ0ioDE8%3D&reserved=0). **(B)** Liver weights from above mentioned mice were measured. **(C)** Representative images of livers. **(D)** Representative images of liver granulomas from infected mice. **(E)** Granuloma size in the liver was determined by manual outlining of the granuloma edge and computer-assisted morphometric analysis. Each data point represents the average granuloma size for an individual mouse, calculated from 5–20 granulomas measured per section. **(F)** Number of eggs per 1.06 mm2 field on H&E-stained liver section at 100X magnification. An average of 25–40 fields per liver section (2 sections per mouse) was assessed. Statistical analysis was performed using ANOVA with Tukey HSD. **(G-H)** Livers from respective groups were homogenized. IL-1β **(G)** and IL-10 **(H)** levels in the homogenates were measured by multiplex assay. Data in **(B)** and **(E-F)** represent pooled results from two independent experiments with 5–8 mice per group. Data in **(G-H)** are representative of two independent experiments of 4–8 mice per group. Bars represent the mean  + /- SD.

We previously reported that the activation of STING by eggs or *S. mansoni* genomic DNA leads to the production of IFN-I, which potently suppresses IL-1β, IL-23, and Th17 cell responses [7]. Additionally, we have shown that STING expression was markedly higher in BL/6 BMDCs as compared to CBA BMDCs [7]. We therefore used CBA mice as a relevant model for this study. To understand whether pharmacological activation of STING reduces proinflammatory cytokine production, we pretreated egg-stimulated BMDCs with diABZI-3, a small molecule, non-nucleotide-based ligand which potently activates STING [16] (Fig 1A, (1)). Due to lower expression of STING, in contrast to BL/6 BMDCs [7], CBA BMDCs expressed less IFN-I in response to eggs but released higher levels of IFNβ when treated with diABZI-3 (Fig 1B). IFNβ production is significantly enhanced when cells were pretreated with diABZI-3 and then stimulated with TLR2 ligand, Pam3CSK4 (Pam3) followed by egg stimulation as compared to BMDCs just stimulated to Pam3 and eggs (Fig 1B). In contrast, IL-1β production was eliminated in these cells (Fig 1C). DiABZI-3 induced significant inhibition of IL-17 in BMDC-T cell cocultures (Fig 1A, (2)), (Fig 1D) and even after 48 h potently induced IFN-I (S1A Fig). Recombinant IFNβ-stimulated cells produced less IL-1β, though the reduction was less pronounced than that observed with diABZI-3 (Fig 1E) suggesting that IFNβ-dependent and independent mechanisms might be responsible for downregulating IL-1β production. We performed dose response analysis to assess the impact of diABZI-3 treatment on IFNβ production (S1B Fig) and cell viability (S1C Fig) in BMDCs. Similar to other studies [17,21], diABZI-3 at the low concentration of 0.1μM, that we used for all in vitro experiments, did not cause cell death in BMDCs (S1C Fig). Given the higher STING expression in BL/6 BMDCs compared with their CBA counterparts [7], pretreatment of BL/6 BMDCs with diABZI-3 followed by egg stimulation resulted in STING overexpression and significantly elevated IFNβ levels (S1D Fig), indicating that the effects of diABZI-3 are strain-specific. The higher levels of IFN-I are inflammatory as previously reported [22–24] and led to higher IL-1β production (S1E Fig). Similar to diABZI-3, another STING agonist, 5,6-dimethylxanthenone-4-acetic acid (DMXAA), was also able to induce IFNβ (S1F Fig) and suppress IL-1β (S1G Fig). Taken together, these results suggest that diABZI-3 induces IFN-I while it suppresses IL-1β and Th17 responses in response to schistosome eggs.

**Fig 3:**
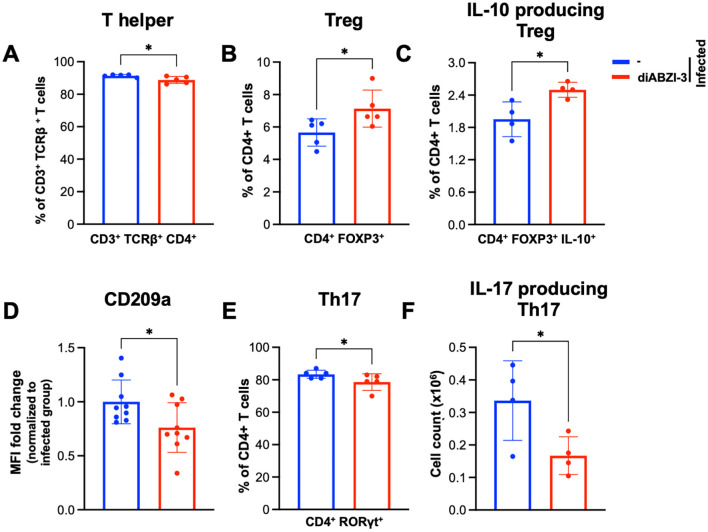
DiABZI-3 treatment reduces Th17 cells but enhances Treg cell recruitment to the *S. mansoni*-infected livers. *S. mansoni* Infected CBA mice were given either no treatment (-) or diABZI-3, and the livers were harvested at week 7 post-infection. Distribution of **(A)** CD45^+^ CD3^+^ TCRβ^+^ CD4^+^, n = 4-5 **(B)** CD45^+^ CD3^+^ TCRβ^+^ CD4^+^ FOXP3^+^, n = 4-5, and **(C)** CD45^+^ CD3^+^ TCRβ^+^ CD4^+^ FOXP3^+^ IL-10^+^, n = 4-5 in the livers of these mice. **(D)** Mean fluorescence intensity (MFI) fold-change of surface CD209a expression (normalized to the infected group) on CD45^+^ CD11c^+^ cells. n = 9. **(E)** Distribution of CD45^+^ CD3^+^ TCRβ^+^ CD4^+^ RORγt^+^ in indicated livers. n = 4-5. **(F)** Quantification of CD45^+^ CD3^+^ TCRβ^+^ CD4^+^ RORγt^+^ IL-17^+^ cells recruited to infected livers. n = 4-5. Data in A, B, C, E, and F are representative of two independent experiments. Data in (D) represents pooled results from two independent experiments with 4-5 mice per group. Bars represent the mean + /- SD.

Type I IFNs are among the first cytokines released by many cells during infection [25] and play crucial roles in the regulation of inflammation [26]. Previous studies suggested the importance of timing and duration of IFN-I administration for the treatment of various diseases [27,28]. To understand whether the timing of diABZI-3 administration could affect its inhibitory impact on IL-1β production, instead of pretreating the cells with diABZI-3 (as shown in Fig 1A, (1)), we first stimulated BMDCs with Pam3/eggs followed by diABZI-3 (Fig 1A, (3)). DiABZI-3 treated cells no longer suppressed IL-1β production (Fig 1F). A time-course analysis at 6, 12, and 24h indicates that diABZI-3 treated cells release high levels of IFNβ as early as 6h, peaking at 12h, which slightly dropped at 24h (S1H Fig). However, this does not suppress IL-1β levels (S1I Fig). Instead, diABZI-3 treated cells exhibit even higher IL-1β levels, especially at 24 h, consistent with previous reports that high levels of IFN-I can promote inflammation [22,29,30]. These results suggest that late administration of diABZI-3 fails to suppress IL-1β production in BMDCs, underscoring the importance of the timing of STING agonist treatment.

### DiABZI-3 reduces egg-induced hepatic immunopathology

We previously reported that STING deficiency results in marked increase in egg-induced granulomatous inflammation [7]. CBA mice were infected with *S. mansoni* intraperitoneally, and diABZI-3 was administered intravenously at the fourth week post-infection, prior to egg deposition by adult worms (Fig 2A). After a 7-week infection, diABZI-3 treated livers were markedly lighter (Fig 2B) and smaller than in the infected only or vehicle control livers (Fig 2C and 2D). On histological H&E examination, the egg granulomas in diABZI-3 treated livers were smaller than infection only livers and vehicle control livers (Fig 2D); the significance of these size differences was confirmed by computer-assisted morphometric analysis (Fig 2E). There were no significant differences in the total number of eggs harbored in the livers of all three groups suggesting that the parasite load was the same (Fig 2F). The diminished granulomatous inflammation in diABZI-3 treated livers was associated with a significant reduction in IL-1β (Fig 2G) but enhancement of IL-10 (Fig 2H) in the liver of these mice. Taken together, these results show that diABZI-3 attenuates inflammatory responses to limit egg-induced hepatic immunopathology.

### DiABZI-3 increases liver regulatory T cells but reduces Th17 cells and CD209a expression

Regulatory T cells (Tregs) are a subset of T cells that play a crucial role in maintaining immune homeostasis by suppressing excessive immune responses during schistosome infection [31]. Type I IFN and STING have been reported to promote the survival and function of Tregs [14,32–34], we thus evaluated whether the administration of diABZI-3 into mice affects the number of Tregs in the liver of *S. mansoni* infected mice. While there were reduced number of total CD4 + T cells in the livers of diABZI-3 treated mice (Fig 3A), we identified a significant increase in the Treg population in the livers of these mice (Figs 3B, S2A). Notably, IL-10 producing Tregs were more frequent in the diABZI-3 treated group (Fig 3C), and consistent with that, there was a significant increase in IL-10 levels in the livers of these mice (Fig 2H). We previously showed that stimulation of C-Type lectin receptor (CLR) CD209a leads to production of IL-1β and the development of pathogenic Th17 cell responses associated with severe egg-induced immunopathology [35]. As previously reported, we found CD209a-expressing cells in the liver granulomas [36]. We also previously showed that STING suppresses CD209a expression and function via IFN-I in BMDCs [7]. We therefore hypothesized that diABZI-3 suppresses CD209a expression in the liver of infected mice and observed a significant decrease in CD209a levels in the livers of diABZI-3 treated mice (Fig 3D), which is associated with diminished granulomatous inflammation (Fig 2E) and reduced IL-1β in the liver of these mice (Fig 2G).

Our previous results established that IL-17 producing CD4^+^ T cells are a major driver of severe pathology in schistosomiasis [37]. Given that diABZI-3 suppresses IL-17 in BMDCs (Fig 1D), we assessed the Th17 cell population in the liver and observed a significant reduction in Th17 recruitment to the livers of diABZI-3 treated mice (Fig 3E). In line with these results, the number of IL-17 producing Th17 cells in these livers were also significantly reduced (Fig 3F), which further explains the smaller granuloma size and reduced immunopathology observed in these mice. This reduction is particularly notable given that high pathology CBA mice typically exhibit significantly elevated CD4^+^ IL-17^+^ T cells in the liver granulomas, spleen, and peripheral blood [38], as well as increased IL-17 protein and mRNA expression in the liver and spleen compared to low pathology BL/6 mice [35]. Taken together, these results suggest that diABZI-3 attenuates egg mediated hepatic immunopathology by increasing the number of IL-10 producing Tregs and suppressing Th17 and CD209a-driven inflammatory responses.

### Blocking STING degradation leads to IL-1β suppression

**Fig 4:**
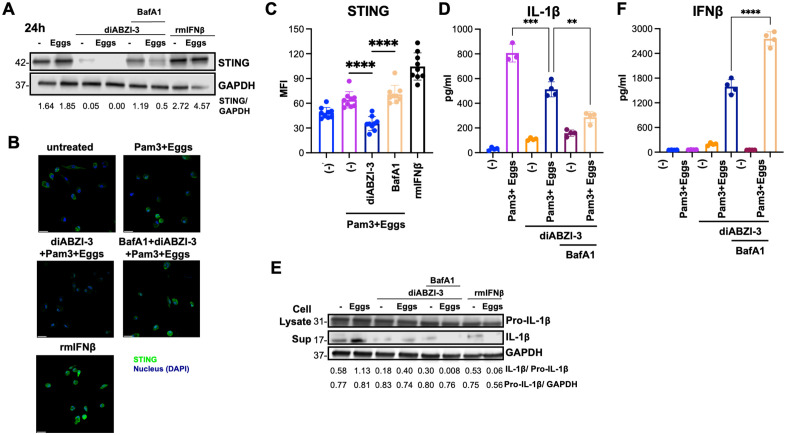
Suppression of V-ATPase activity results in blockade of STING degradation. (A) 1 × 10^6^ CBA BMDCs were plated in twelve-well plates and pretreated with 0.1 µM diABZI-3 for 2h, Pam3 for 1h, and stimulated with eggs per well for 24h. Some cells were stimulated with 250nM BafA1 and diABZI-3 simultaneously, and some cells were pretreated with 1000U rmIFNβ. Cell lysates were used for western lot analysis using Abs against STING and GAPDH. **(B)** Representative confocal microscopy images of BMDCs treated as described in **A.** Cells were stained for STING (green), and nuclei (blue) were counterstained with DAPI. Fields are representative of at least ten fields of view and two independent experiments. Scale bar: 20μm. **(C)** Quantification of STING MFI. Individual data points represent the mean intensity of one cell. Data were analyzed by one-way ANOVA with Bartlett’s correction and Tukey multiple comparisons. **(D)** IL-1β and **(F)** IFNβ in cell culture supernatant was measured by ELISA. **(E)** Immunoblot analysis of CBA BMDCs treated as described in A. Cell lysates and supernatant were used for western blot analysis using Abs against IL-1β and GAPDH. Results are representative of two independent experiments. Bars represent the mean + /- SD.

While STING/IFN-I has a protective role in schistosomiasis, sustained STING activation can lead to inflammation. In fact, excessive levels of double-stranded DNA (dsDNA) can result in autoinflammatory diseases, such as systemic lupus erythematosus (SLE) [39,40]. STING post-Golgi trafficking to the recycling endosomes and further fusion with the lysosomes has been reported by multiple groups to be critical for its degradation and signaling termination [8,41–44]. We hypothesized that blocking STING degradation would enhance STING signaling and hence IL-1β suppression. This was confirmed as diABZI-3 treated BMDCs triggered complete STING degradation 24h post-treatment, but this was blocked by bafilomycin A1 (BafA1), indicating that acidification of endosomes is important since BafA1 suppresses V-ATPase activity and disrupts acidification of endosomes/lysosomes (Figs 4A, S3A complete blot, S3B 2h post-treatment, S3C). Consistent with this data, using confocal microscopy, we observed almost complete degradation of STING in diABZI-3 treated cells (Fig 4B, middle left panel). BafA1-pretreated BMDCs exhibit significantly higher STING expression, further confirming that BafA1 retains STING in the endosomes and prevents its lysosomal degradation (Fig 4B, middle right panel). BMDCs treated with recombinant murine IFNβ (rmIFNβ) expressed high levels of STING (Fig 4B, bottom panel), as STING is an Interferon-stimulated gene (ISG) [45] and serves as a positive control, as STING in these cells did not undergo degradation (Fig 4B, quantifications shown in 4C). Blockade of STING degradation and hence retention of STING led to a dramatic reduction of mature IL-1β (Fig 4D; total IL-1β shown in Fig 4E). Recombinant murine IFNβ also induced a significant decrease in IL-1β expression (Fig 4E). Additionally, the blockade of STING degradation via BafA1 further enhanced IFNβ expression (Fig 4F). These data supported a model where STING degradation involves acidified endolysosomes and blocking its degradation leads to IL-1β suppression.

### DiABZI-3 treated mice develop reduced egg-induced intestinal immunopathology, increased regulatory T cells but reduced Th17 cells and CD209a expression

**Fig 5:**
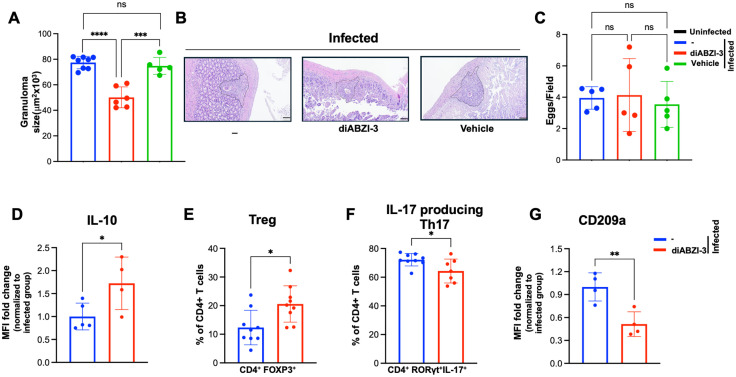
diABZI-3 treatment mitigates inflammation in the intestine. Intestines from diABZI-3 treated and untreated *S. mansoni* infected mice were harvested at 7 weeks post-infection. **(A)** Granuloma size in the intestines was determined by manual outlining of the granuloma edge and computer-assisted morphometric analysis. Each data point represents the average granuloma size of 7-–10 granulomas in two sections from an individual mouse. **(B)** Representative images of intestinal granulomas from infected mice. **(C)** Number of eggs per 1.06mm2 field on H&E-stained liver section at 100X magnification. An average of 10 fields per liver section (2 sections per mouse) was assessed. **(D)** MFI fold-change of IL-10 expression in CD45^+^ CD3^+^ TCRβ^+^ CD4^+^ cells, n= = 4-–5. **(E)** Distribution of CD45^+^ CD3^+^ TCRβ^+^ CD4^+^ FOXP3^+^ and **(F)** of CD45^+^ CD3^+^ TCRβ^+^ CD4^+^ RORγt^+^ IL-17^+^, n = 8–9. **(G)** MFI fold-change of surface CD209 expression on CD45^+^ CD11c^+^ cells, n = 4. Data in (A), (C), (D), and (G) are representative of two independent experiments. Data in (E) and (F) represent pooled results from two independent experiments with 4–5 mice per group. Bars represent the mean  +/- SD.

We studied the impact of diABZI-3 in the gut during schistosome infection, a model of intestinal inflammatory disease, which is associated with alterations of gut microbiome. Morphometric analysis of single-egg granulomas suggested that the average intestinal granuloma size was significantly smaller in diABZI-3 treated mice as compared to infected only and vehicle control groups (Fig 5A and 5B), however, the number of eggs present in the intestines did not significantly differ between the mouse groups, indicating that the parasite load did not associate with the severity of pathology (Fig 5C). Consistent with hepatic findings (Fig 2), IL-10 producing CD4 T cells were more frequent in the diABZI-3 treated intestines (Fig 5D). We also observed a significant increase in the Treg population in the intestines of the diABZI-3 treated mice (Fig 5E). In contrast, the IL-17 producing Th17 cells were significantly diminished in these mice (Fig 5F). Furthermore, similar to hepatic findings (Fig 3D), we observed reduced CD209a levels in the intestines of diABZI-3 treated mice (Fig 5G). Collectively, these findings demonstrate that diABZI-3 attenuates egg-induced immunopathology in the intestine by enhancing Treg population and suppressing Th17- and CD209a-mediated granulomatous inflammation.

### DiABZI-3 partially ameliorates infection-induced gut dysbiosis

**Fig 6:**
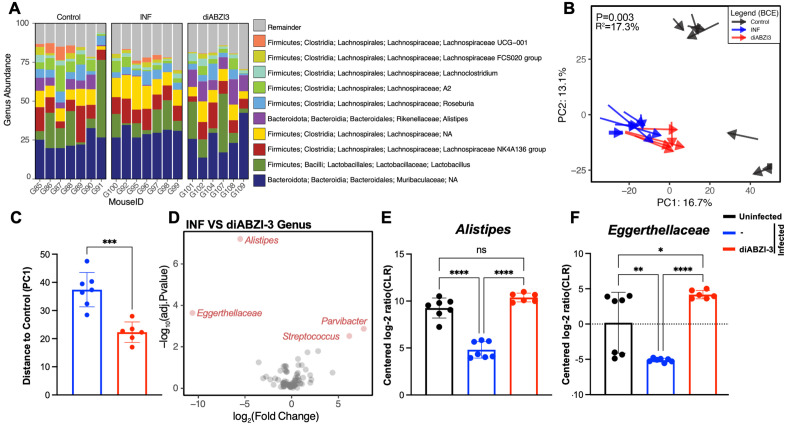
DiABZI-3 treatment restored the microbiome of *S. mansoni*-infected mice to a composition similar to uninfected controls. CBA mice were infected with *S. mansoni*, injected with respective treatments, and sacrificed at 7 weeks post-infection. Fecal samples were harvested for **(A)** 16S RNA sequencing, obtaining 193,058 ± 32,040 reads per sample post processing (mean±SD). Genus-summarized taxonomic abundances at 7 weeks post-infection. **(B)** Microbiome similarity was determined through principal coordinate analysis of animals at 4 and 7-week time points. PCoA ordination was conducted with Aitchison distance with samples from the same mouse represented as a vector. R2 = 0.17l P = 0.003, Statistical analysis by PERMANOVA. (C) diABZI3-treated animals are more similar in composition to uninfected controls than untreated animals, as calculated by the average distance on the first principal coordinate (PC1). Statistical testing was done by Welch’s t-test. **(D)** Differential genus abundance of 7-week post-infection animals determined by ALDEx2 [47]. Significantly different genera are marked in red (FDR corrected Welch’s t test < 0.1). Abundances of *Alistipes* (E) and *Eggerthellaceae* (F) in microbiome samples from each group. Statistical testing was performed by ANOVA with Tukey HSD on the Centered log-2 ratio (CLR) transformed abundance. Bars represent the mean + /- SD.

To understand the role of diABZI3 in regulating intestinal inflammation during schistosome infection, we performed 16S rRNA sequencing on fecal samples collected before and after diABZI-3 treatment in *S. mansoni* infected CBA mice. Post-infection, while the major families of the *Muribaculaceae* and *Lactobacillus* spp. remained unchanged, specific clades displayed evidence of differential abundance between groups (Fig 6A). As expected, microbiomes from diABZI-3 treated animals more closely resembled those from uninfected animals of which *Alistipes* spp., a bacterial genus often associated with protection against inflammatory diseases such as colitis, was a striking example being depleted in infected mice but preserved in diABZI-3 treated [18,19,46]. These observations were further supported by principal coordinates analysis (PCoA) which demonstrated that while infection strikingly reshaped gut microbiota composition (Fig 6B), diABZI-3 treated animals were more similar in composition to controls (Fig 6C). To more carefully identify microbes which were differentially abundant as a function of diABZI-3 treatment in infected animals, we performed a univariate compositionally aware analysis identifying 4 significantly different clades (Fig 6D). Two of the microbes which were increased in abundance in diABZI-3 treated mice were the previously identified *Alistipes* and *Eggerthellaceae*, while infected mice had higher levels of *Parvibacter* and the inflammatory *Streptococcus* spp. *Alistipes* and *Eggerthellaceae* were found to be reverted to uninfected levels, which is of note given that these species have been previously positively associated with recovery from colitis and suppression of Th17 cells (Fig 6E and 6F) [20]. Taken together, these results suggest that diABZI-3 treatment shifts the gut microbiome of *S. mansoni* infected mice toward a profile resembling that of uninfected controls.

## Discussion

Our results demonstrate that the STING agonist diABZI-3 potently induces IFN-I production while suppressing proinflammatory IL-1β and Th17 responses in BMDCs, leading to reduced hepatic and intestinal granulomatous inflammation in vivo. These observations align with our previous findings that STING plays a protective role in modulating egg-induced immunopathology by skewing immune responses toward an anti-inflammatory Th2 environment [7]. The current study extends these insights by elucidating the molecular mechanisms underlying STING agonist-mediated protection and highlights the critical importance of timing in therapeutic administration.

A key observation from our in vitro experiments is that diABZI-3 pretreatment of BMDCs significantly enhances IFNβ production and abolishes IL-1β expression in response to schistosome egg stimulation (Fig 1). This effect is specific, as demonstrated by the lack of suppression when diABZI-3 is administered after egg stimulation (Fig 1), suggesting that early IFN-I signaling is crucial for dampening proinflammatory cascades. The failure of late diABZI-3 administration to suppress IL-1β, coupled with elevated IL-1β levels in these conditions, points to a potential proinflammatory role of sustained or delayed IFN-I signaling, as previously reported in contexts of excessive type I IFN production [22]. This finding has important implications for the clinical application of STING agonists, emphasizing the need for precise timing to avoid exacerbating inflammation. In the absence of early IFN-I, the accumulation of IL-1β and IFNβ may contribute to a cytokine storm, a phenomenon observed in various inflammatory diseases [27]. Future studies should explore the optimal therapeutic window for STING agonist administration to maximize anti-inflammatory effects.

The in vivo experiments further validate the protective role of diABZI-3, as a single dose administered at week 4 of *S. mansoni* infection significantly reduced liver granuloma size and IL-1β levels. This reduction in immunopathology is likely driven by STING-mediated IFN-I production, which leads to suppression of IL-1β production, thereby limiting Th17 cell activation [7]. These results highlight the potential of STING agonists as adjunctive therapies to complement existing treatments like praziquantel, which fail to address immunopathology or prevent reinfection [2].

Activation of STING via STING agonists has been shown to be a promising strategy to treat cancers and viral and autoimmune diseases [17,48–51]. Use of STING agonists such as diABZI-3 has many advantages over IFN therapy, including higher stability, being efficient at lower dose and storage at room temperature. Additionally, treatments with recombinant IFN-I have shown to have side effects and mixed results [52]. While diABZI-3 is generally stable, but similar to the other STING agonists has a transient nature in terms of inducing STING activation [8,48]. Similar to previous findings [8], in this study we showed that blocking STING degradation can lead to sustained STING signaling. This feature may have therapeutic implications in infectious diseases and cancer. This study also identified the motif (helix aa281-297) critical for STING degradation [8] which is found to contain mutations associated with the disease SAVI [53], suggesting that sustained activation of STING might lead to high levels of IFN-I and inflammation in these patients. Blocking STING degradation with bafilomycin A1 prevented lysosomal acidification and sustained STING expression, leading to a dramatic reduction in IL-1β expression. This finding suggests that strategies to stabilize STING signaling could amplify the anti-inflammatory effects of agonists such as diABZI-3. However, sustained STING activation must be approached cautiously, as excessive STING signaling has been implicated in autoinflammatory diseases such as systemic lupus erythematosus [54]. The balance between prolonged STING activation and potential proinflammatory consequences warrants further investigation.

The reduced recruitment of Foxp3^+^ Tregs into infected organs has also been reported in other infection models [55]. The diABZI-3-induced increase in Tregs in the liver and intestine is particularly noteworthy, as we previously demonstrated lower STING/IFN-I expression in BMDCs from CBA mice compared to BL/6 mice [7]. Treg cells have been shown to be regulated by IFN-Is in the intestine. Lack of IFNAR leads to reduction of Treg in the intestine, resulting in the enhancement of Th1 and Th17 effector cells in the colon [56]. Another study showed that frequency of Treg was reduced in colonic lamina propria of STING deficient mice [14], but to our knowledge, no study has demonstrated that STING agonist administration leads to the expansion of Tregs and suppression of Th17 cells in liver and intestine tissues. These findings are significant, as they may represent novel therapeutic targets for modulating liver and intestine inflammation.

Supporting our findings, multiple studies link *Alistipes* and *Eggerthellaceae* species to beneficial immunomodulatory effects [57,58]. Oral administration of *Alistipes* ameliorated DSS-induced colitis in mice by reducing intestinal epithelial damage in an IL-10 dependent manner [19]. Another study demonstrated NOD2 knockout mice had an enrichment of *Alistipes*, anti-inflammatory cytokines, TGFβ and IL-10, and CD4^+^LAP^+^FoxP3^+^ regulatory T cells [59]. Similarly, members of the *Eggerthellaceae* family, such as *Eggerthella* lenta have been implicated in reducing Th17 differentiation [20]. The enrichment of *Alistipes* and *Eggerthellaceae* spp., along with the IL-10 increase and anti-inflammatory profile observed following diABZI-3 treatment may therefore reflect a shift toward a microbiome composition that is less permissive to Th17 driven pathology. In the context of schistosomiasis, where hepatic granuloma formation and gut epithelial barrier disruption drive a proinflammatory milieu, enrichment of these taxa could contribute to the attenuation of intestinal inflammation observed with diABZI-3. While the restoration of *Alistipes* and *Eggerthellaceae* following diABZI-3 treatment is associated with an anti-inflammatory microbiome profile, we acknowledge that these associations are correlative in nature. Functional validation using germ-free colonization models or metabolite specific interventions will be necessary to establish a causal relationship between these microbial shifts and the modulation of Th17 driven intestinal pathology.

Given the global burden of schistosomiasis and the limitations of current treatments, STING agonists represent a promising strategy to reduce disease severity and improve patient outcomes. Future research should focus on optimizing dosing regimens, evaluating long-term safety, and testing these findings in preclinical models that more closely mimic human disease.

## Materials and methods

### Ethics statement

All mice were maintained at the Pennsylvania State University’s Animal Facilities in accordance with the Institutional Animal Care and Use Committee (IACUC) protocol number 202102124 and the Association for Assessment and Accreditation of Laboratory Animal Care guidelines.

### Mice, parasites, and infection

Six- to eight-week-old female CBA/J and C57BL/6 mice were purchased from The Jackson Laboratory and Swiss Webster mice from Taconic Biosciences. At least five mice per experimental group were infected by i.p. injection with 80 *Schistosoma mansoni* (PR-1) cercariae. *Schistosoma mansoni* cercarias shed from infected Biomphalaria glabrata (M-line) snails were provided by the Schistosomiasis Resource Center of the Biomedical Research Institute (Rockville, MD) through NIH-NIAID Contract HHSN272201700014I (to PK). Live schistosome eggs were isolated from livers of infected Swiss Webster mice under sterile conditions by a series of blending and straining techniques, as previously described [60]. All infected mice were sacrificed at seven weeks of post-infection. Livers and intestines were harvested and further processed for histopathology, flow cytometry, and immunological analyses.

### Reagents

RPMI 1640 medium and penicillin-streptomycin were obtained from Cytiva, FBS from Atlas Biologicals, glutamine from Gibco, recombinant murine GM-CSF from PeproTech, Red Blood Cell Lysing Buffer, 2-mercaptoethanol, Bafilomycin A1, Percoll, Hank’s balance set solution (HBSS) from Sigma, Recombinant murine IFNβ (rm IFNβ) from PBL Assay Science, DMXAA, diABZI (compound 3) from Sellekchem (for in vitro experiments) and Invivogen (for in vivo experiments) LPS, Pam3CSK4, ProLong Diamond Antifade Mountant with DAPI and EBioscience FOXP3/Transcription Factor Staining Buffer Set from Invitrogen, Cell Counting Kit-8 (CCK-8) cell viability assay from Dojindo, Kumamoto, Japan, BD GolgiStop Protein Transport Inhibitor (containing Monensin) from BD Biosciences.

### Cells, cell cultures and cell stimulations

***DC Cultures*** BMDCs were generated as described previously [35]. 1x10^5^ BMDCs were plated and stimulated with 100 eggs/1x10^5^ BMDCs. Some cells were stimulated with Pam3CSK4 (1µg/ml) for 1h followed by eggs for 24h (unless indicated otherwise the figure legend).

***DC-T Cell Co-cultures*** CD4^+^ T cells were prepared from normal CBA spleens using a CD4^+^ T Cell Isolation Kit II for mouse (Miltenyi Biotec, Cambridge, MA) in accordance with the manufacturer’s instructions. Purified CD4^+^ T cells (2x10^5^) were cultured with 10^5^ syngeneic BMDCs and stimulated with 100 eggs plus 8 × 10^4^ anti-CD3/CD28 coated beads (Gibco Dynal Dynabeads) for 48h.

### ELISA

Primary BMDCs were generated as described above. Cytokine protein measurements were performed in culture supernatants for IL-1β, IL-17A, IFNβ and TNFα, and in tissue homogenates for IL 1β and IL-10 using ELISA kits from R&D Systems in accordance with the manufacturer’s instructions.

### Cell viability assay

Cell viability of different concentrations of diAZBI-3 was measured using Cell Counting Kit-8. The assay was conducted following the manufacturer’s instructions. Cell viability was assessed by measuring the absorbance (450nm) using a plate reader.

### Western blotting

BMDCs were washed, lysed, and prepared with Laemmli Buffer (BioRad). Samples were run on an SDS-PAGE gel and transferred to a nitrocellulose membrane (BioRad), which was blocked in 5% BSA. Protein expression was detected with Antibodies specific for STING (Novus Biologicals), IL-1β (Cell Signaling), and GAPDH (Cell Signaling).

### Liver and intestine histopathology

After 7 weeks of infection, all mice were sacrificed and examined for hepatic and intestinal immunopathology. Liver and intestine samples obtained from the infected mice were fixed in 10% buffered formalin, processed and sectioned using standard histopathological techniques, stained with hematoxylin and eosin (H&E), and examined by light microscopy. Individual granulomas containing a single egg were measured by manual outlining of the granuloma edge and computer-assisted morphometric analysis (cellSens software, Olympus). Dispersed liver cells were prepared and stained for flow cytometry as fully described under “Flow cytometry”.

### Liver dissociation

Liver tissue was dissected with a razor blade and treated with Liberase (30µg/ml) and DNAse (20U/ml) in 5mL RPMI for 25 minutes in a 37 °C incubator and homogenized to a single cell suspension by mechanical dissociation using a 3ml syringe and 70-µm cell strainer. Single cell suspensions were washed in 2mM EDTA with 2% FBS prior to isolation of non-parenchymal cells (NPCs) through repeated low-speed centrifugation (three rounds of 50g x 3 minutes) where NPCs remain in the supernatant. Red blood cells were lysed prior to quantification and staining for flow cytometry with red blood cell lysis buffer.

### Lamina propria cell isolation

Cells were isolated from lamina propria by dissecting colons from mice. Feces were removed, the colons were opened longitudinally and washed with 1x HBSS with 2% FBS, then cut into small pieces. Colon fragments were transferred to a 50 mL conical tube with pre-warmed EDTA digestion buffer (1xCMF-HBSS with 10% FBS and 5 mM EDTA) and incubated for 30 min in a 37^o^C shaking incubator at 250 rpm to remove epithelium. Tissue fragments were then washed with RPMI and dissociated again with collagenase digestion buffer (RPMI with 5%FBS and 1mg/ml collagenase type H) in a 37 °C shaking incubator with 250 rpm for 1.5h. Cells were passed through a 70 μm cell strainer and resuspended in 80% Percoll buffer (10XHBSS with 80% Percoll and 10% cell culture grade water). Percoll gradient was made by slowly passing cell suspension through a glass Pasteur pipette into a tube containing 40% Percoll (RPMI with 2%FBS and 40% Percoll). Lamina propria cells were then centrifuged at 21^o^C and 860 × g for 20 min, transferred to a new 15ml conical tube, and gently mixed with cold 1xHBSS with 2% FBS to remove Percoll. Cells were centrifuged at 4 °C at 600 × g for 5 min and resuspended in cRPMI containing GolgiStop Protein Transport Inhibitor and Brefeldin A in a 37 °C incubator for 4 h. Cells were quantified for flow cytometry antibody staining.

### Flow cytometry

Single cell suspensions from livers and intestines were washed in PBS prior to viability staining (Live Dead Near IR) and staining for surface proteins for 30 minutes. Excess antibody washed out with PBS prior to sample fixation. Antibodies for surface staining were used for 30 minutes on ice. EBioscience FOXP3/Transcription Factor Staining Buffer Set was used for intracellular and intranuclear staining in accordance with the manufacturer’s instructions. Samples were analyzed using a Cytek Aurora (4 lasers). Antibodies used for flow cytometry included the following: eFluor 506 anti-mouse CD45 (Invitrogen, 69-0451-82), RB744 anti-mouse TCRb (BD, 757028), cFluor B515 anti-mouse CD3 (Cytek, R7-20717), Spark NIR 685 anti-mouse CD4 (Biolegend, 100476), eBioscience APC anti-mouse FOXP3 (Invitrogen, 17-5773-82), CD209a (BD, 740621), eFluor450 anti-mouse CD11c (Thermo Fisher, 48-0114-82), BV785 anti-mouse IL-17 (Biolegend, 506928), BV711 anti-mouse IL-10 (Biolegend, 50504), PE anti-mouse RORγT (Thermo Fisher, 12-6981-82) and live/dead near-IR dead cell stain kit (Invitrogen, L34975).

### Confocal microscopy

Confocal microscopy was performed on a Leica Stellaris confocal laser-scanning microscope. Cells were stained with STING utilizing primary antibody (Novus Biologicals, NBP2-24683) and secondary antibody (abcam, ab150077). LASX software (v3.10.0) was used to measure fluorescence intensity via the quantify histogram function for all images. An ROI polygon was drawn around each cell individually and the pixel intensity of the appropriate detector channel for STING within the ROI is averaged according to ROI pixel area and reported as mean intensity in LASX. Abundance of STING was quantified from 3-5 cells per at least 3 fields of view for each treatment.

### Microbiome characterization

Microbiome high depth V4 16S rRNA amplicon sequencing was performed using standard protocols in the One Health Microbiome Center Co-laboratory at Pennsylvania State University. Complete protocols are available at https://github.com/BisanzLab/OHMC_Colaboratory. Briefly, DNA was extracted using Qiagen DNeasy Powersoil Pro Kits following the manufacturer’s instructions with bead lysis performed using a Qiagen Tissuelyzer III. Liquid handling was performed by Integra mini-96 and a QIAcube HT. Primary PCR was conducted using 515F and 806R primers with partial overhangs for i7 and i5 adapters using KAPA HiFi hot start enzymes and SYBR green to monitor amplification progress in real time (QIAquant 384). Resulting amplicons were diluted 100X and indexed using 10nt unique dual indexes based on the Illumina IDT Tagmentation Set ABCD before quantification with PicoGreen (Life Technologies) and pooling at equal molar concentrations. The final library underwent two rounds of size selection using Ampure XP beads before sequencing on an Illumina Nextseq 2000 (PSU Genomics Research Incubator) using a XLEAP P1 600 cycle flow cell.

Raw sequencing data were processed using Quantitative Insights into Microbial Ecology 2 (QIIME2) version 2023.5 [61]. Cutadapt was used to remove primer sequences allowing for a 15% error rate. Samples without a valid primer sequence were removed. Based on read quality, forward reads were truncated at 220 bases and reverse reads at 150 bases. DADA2, as implemented in QIIME2 [62], was applied to denoise the data, merge paired reads, remove chimeric sequences, and resolve amplicon sequence variants (ASVs). Taxonomic classification of representative sequences was performed using DADA2’s classifier with the SILVA 138 database for 515F/806R sequences [63]. ASVs corresponding to mitochondrial or chloroplast sequences were excluded from downstream. Two positive controls (Zymo D6302), and 4 negative controls were included in the sequencing run. Negative controls obtained <87 reads/sample of probable cross-contamination origin. No reads belonging to strains not found in the positive control were observed. Experimental samples contained 193,058 ± 32,040 reads post-processing. Data processing and visualization were performed using qiime2R v 0.99.6. Diversity analyses were performed using Vegan v 2.7-1, and differential abundance calculations were performed using ALDEx2 v1.40.0.

### Statistics

Student’s *t* test was performed for statistical significance using GraphPad Prism10 unless specified. P values ≤0.05 were considered significant. Student’s t-test was performed unless otherwise specified. All data are shown as mean ± SEM.

## Supporting information

S1 Fig (A) DiABZI-3 induced IFN-I after 48h. DiABZI-3 treated BMDCs from CBA mice were cultured with CD4^+^ naive T cells isolated from CBA mice spleens for 48 h. IFNβ in the supernatants was measured by ELISA. **DiABZI-3 at the concentration of 0.1μM did not cause cell death in BMDCs**. BMDCs from CBA mice were stimulated with various concentrations of diABZI-3. IFNβ (B) and cell viability (C) were measured. **Pretreatment of BL/6 BMDCs with diABZI-3 followed by egg stimulation resulted in elevated IFN-I and IL-1β levels**. BMDCs from C57BL/6 mice were pretreated with diABZI-3 for 2 h followed by Pam3 for 1h and eggs (schematic in Fig. 1A(1)). IFNβ (D) and IL-1β (E) were measured by ELISA. **STING agonist DMXAA induces IFNβ and suppresses IL-1β production**. BMDCs were pretreated with 100µg/ml of DMXAA for 2h followed by stimulation with LPS for 1h and then eggs for 24h. IFNβ (F) and IL-1β (G) in the supernatants were measured by ELISA. **Late administration of diABZI-3 does not suppress IL-1β production in BMDCs**. BMDCs were plated in 96-well plate and stimulated with Pam3, eggs and diABZI-3 as shown in Fig. 1A (3) for 6, 12, or 24h. IFNβ (H) and IL-1β (I) were measured by ELISA. Bars represent the mean + /- SD cytokine levels of three biological replicates from one representative experiment of two experiments with similar results. For this and all figures: * p ≤ 0.05, ** p ≤ 0.005, *** p ≤ 0.0005, **** p ≤ 0.00005.
(TIFF)

S2 Fig Gating strategy of regulatory T cells. (A) Representative flow cytometric panels for CD45^+^ CD3^+^ TCRb^+^ CD4^+^ FOXP3^+^.
(TIFF)

S3 Fig (A) diABZI-3 induced complete STING degradation at 24h post-treatment. Immunoblot analysis of 1 × 10^6^ CBA BMDCs plated in twelve-well plates and pretreated with diABZI-3 for 2h, Pam3 for 1h and stimulated with eggs for 24h. Some cells were stimulated with rmIFNβ. (B) **diABZI-3 triggered STING degradation in BMDCs as early as 2h post-treatment.** The same experiment as explained in A, except that cells were incubated for 2h after stimulation with 250nM BafA1 and diABZI-3 simultaneously. Cell lysates in (A) and (B) were used for western blot analysis using Abs against STING and GAPDH. (C) **Schematic illustration of suppression of STING degradation by BafA1 in endosomes.** Data represent one representative experiment of two experiments with similar results.
(TIFF)

S1 Data Raw data value. (XLSX)

S1 File Raw images. (PDF)
