# Supplementary figures and images for: STING agonist protects against exacerbation of schistosome egg-induced immunopathology
Source: PLoS Pathog. 2026 Jul 10;22(7):e1014394. doi: 10.1371/journal.ppat.1014394 (PMC13353948; doi:10.1371/journal.ppat.1014394)

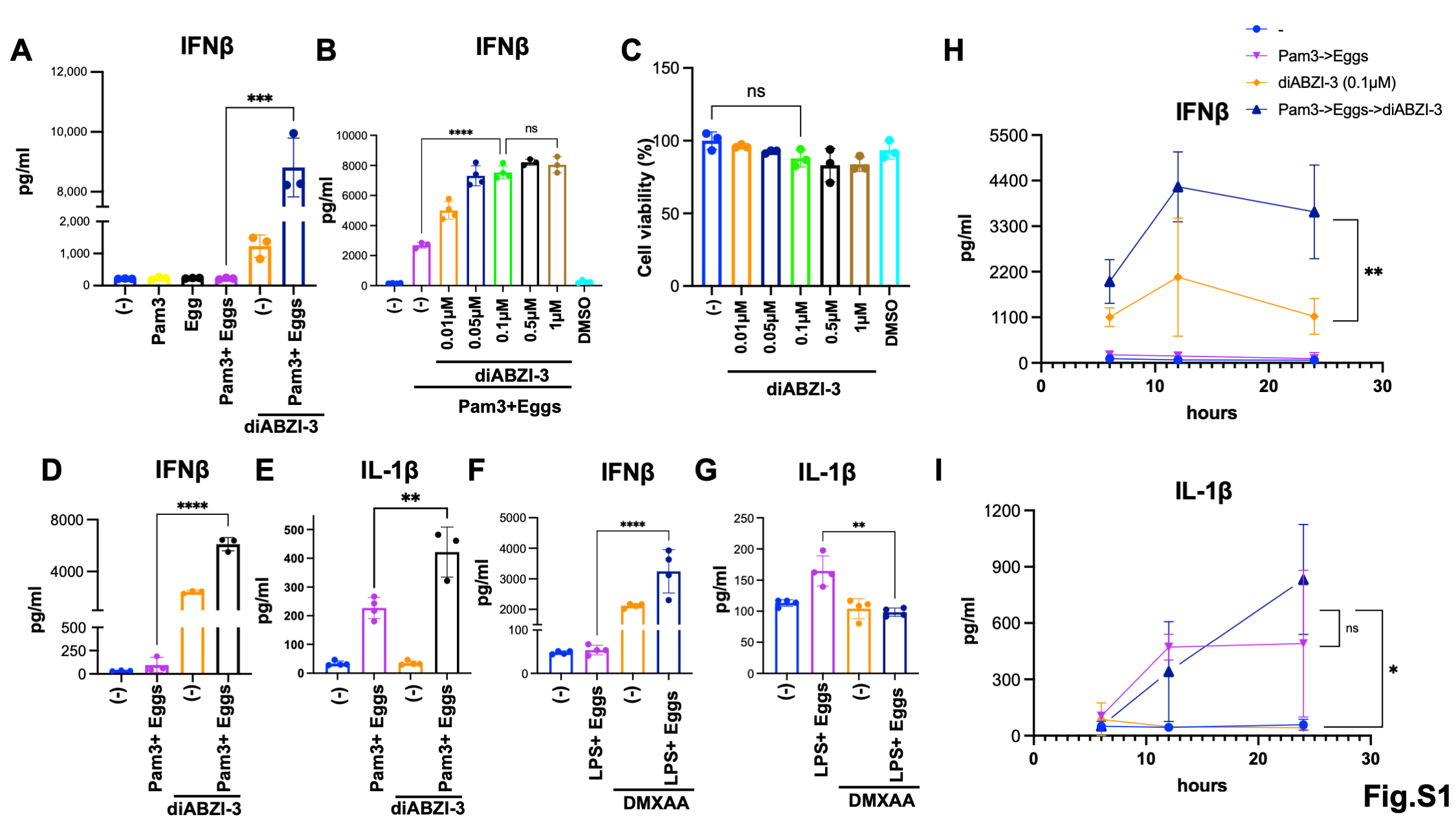

Supplement: S1 Fig — DiABZI-3 treated BMDCs from CBA mice were cultured with CD4+ naive T cells isolated from CBA mice spleens for 48 h. IFNβ in the supernatants was measured by ELISA. DiABZI-3 at the concentration of 0.1μM did not cause cell death in BMDCs. BMDCs from CBA mice were stimulated with various concentrations of diABZI-3. IFNβ (B) and cell viability (C) were measured. Pretreatment of BL/6 BMDCs with diABZI-3 followed by egg stimulation resulted in elevated IFN-I and IL-1β levels. BMDCs from C57BL/6 mice were pretreated with diABZI-3 for 2 h followed by Pam3 for 1h and eggs (schematic in Fig. 1A(1)). IFNβ (D) and IL-1β (E) were measured by ELISA. STING agonist DMXAA induces IFNβ and suppresses IL-1β production. BMDCs were pretreated with 100µg/ml of DMXAA for 2h followed by stimulation with LPS for 1h and then eggs for 24h. IFNβ (F) and IL-1β (G) in the supernatants were measured by ELISA. Late administration of diABZI-3 does not suppress IL-1β production in BMDCs. BMDCs were plated in 96-well plate and stimulated with Pam3, eggs and diABZI-3 as shown in Fig. 1A (3) for 6, 12, or 24h. IFNβ (H) and IL-1β (I) were measured by ELISA. Bars represent the mean + /- SD cytokine levels of three biological replicates from one representative experiment of two experiments with similar results. For this and all figures: * p ≤ 0.05, ** p ≤ 0.005, *** p ≤ 0.0005, **** p ≤ 0.00005. (TIFF) [file ppat.1014394.s001.tiff]

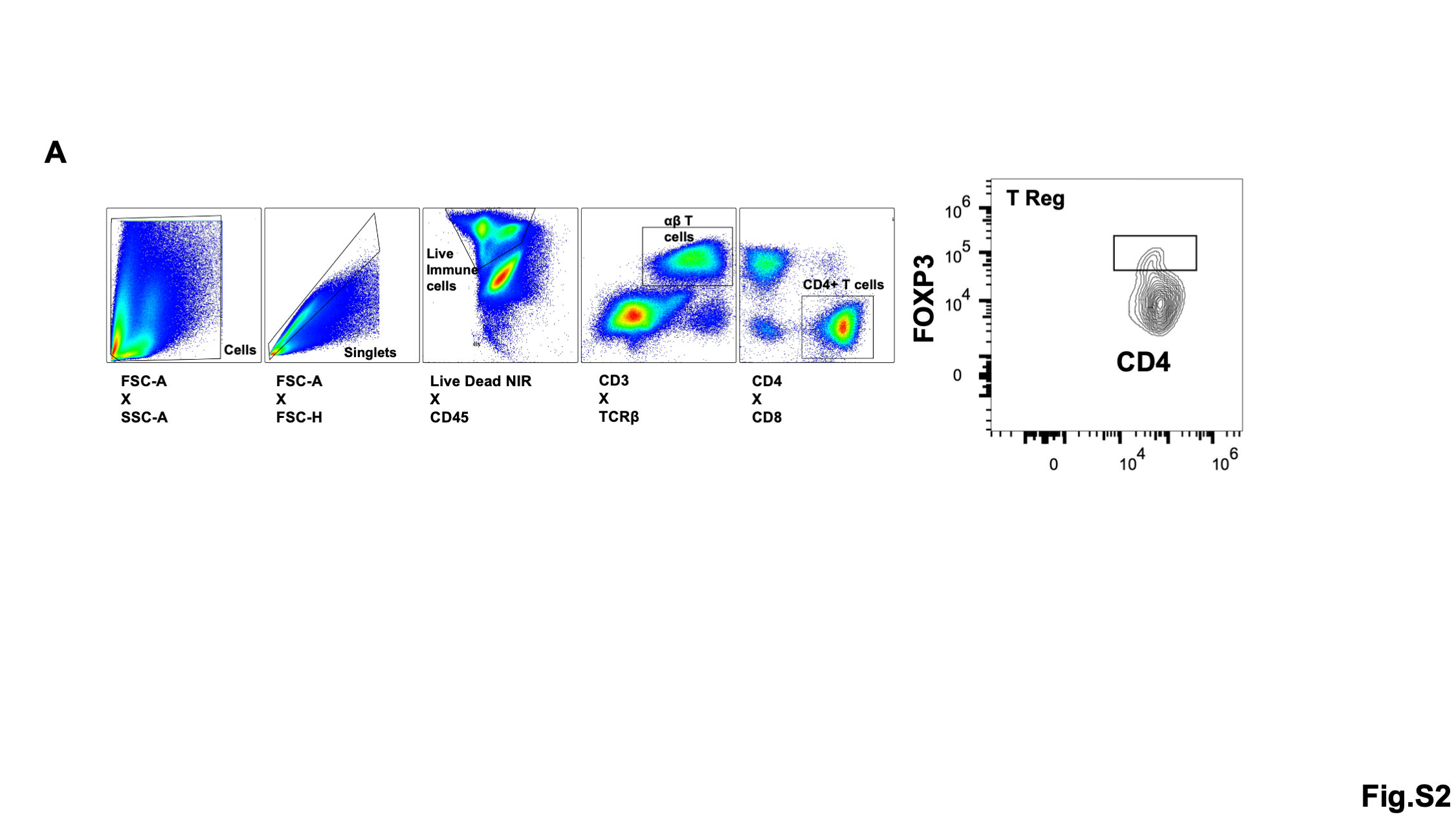

Supplement: S2 Fig — (A) Representative flow cytometric panels for CD45+ CD3+ TCRb+ CD4+ FOXP3+. (TIFF) [file ppat.1014394.s002.tiff]

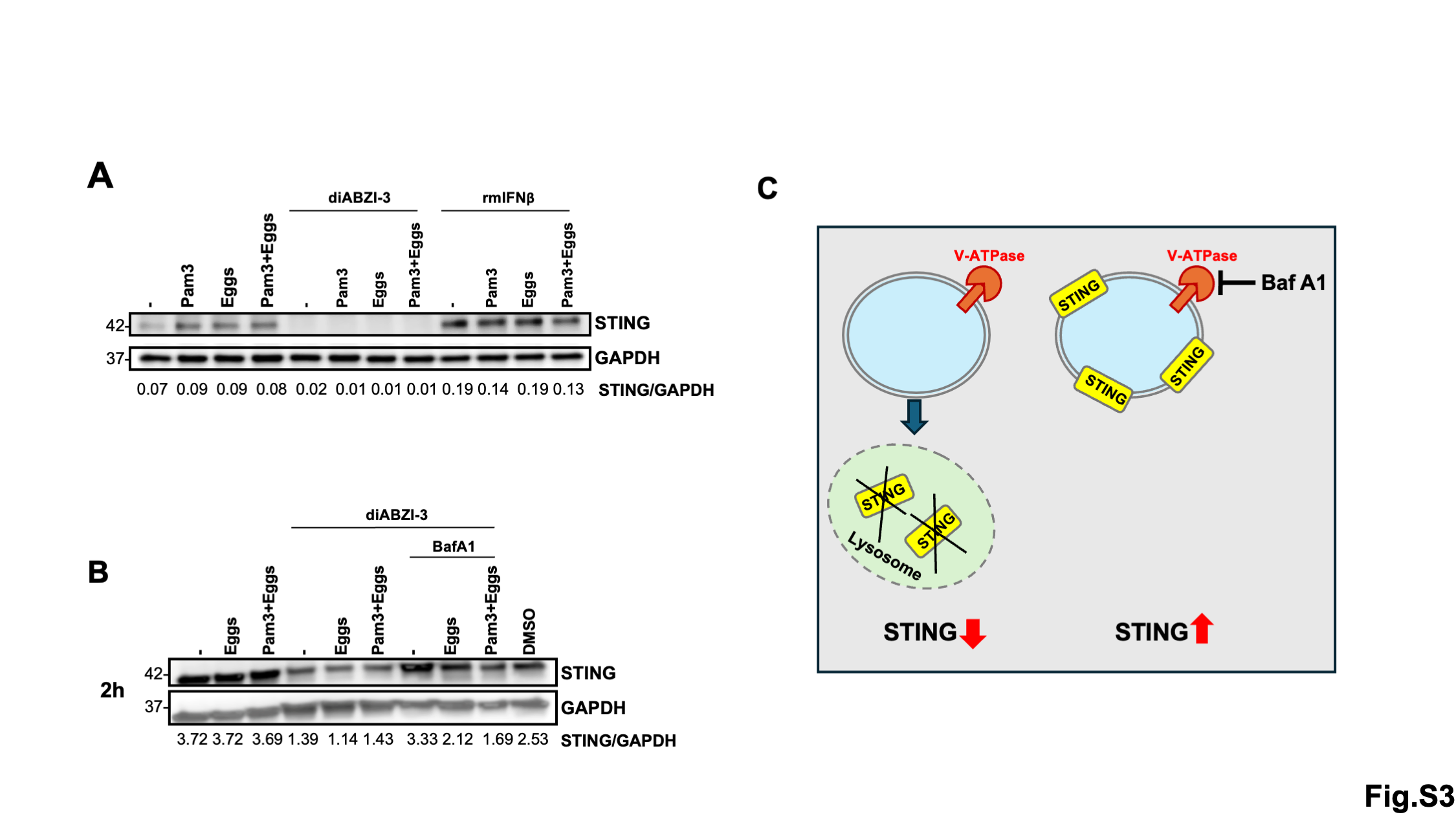

Supplement: S3 Fig — Immunoblot analysis of 1 × 10^6^ CBA BMDCs plated in twelve-well plates and pretreated with diABZI-3 for 2h, Pam3 for 1h and stimulated with eggs for 24h. Some cells were stimulated with rmIFNβ. (B) diABZI-3 triggered STING degradation in BMDCs as early as 2h post-treatment. The same experiment as explained in A, except that cells were incubated for 2h after stimulation with 250nM BafA1 and diABZI-3 simultaneously. Cell lysates in (A) and (B) were used for western blot analysis using Abs against STING and GAPDH. (C) Schematic illustration of suppression of STING degradation by BafA1 in endosomes. Data represent one representative experiment of two experiments with similar results. (TIFF) [file ppat.1014394.s003.tiff]
