# Supplementary material for: STING agonist protects against exacerbation of schistosome egg-induced immunopathology
Source: PLoS Pathog. 2026 Jul 10;22(7):e1014394. doi: 10.1371/journal.ppat.1014394 (PMC13353948; doi:10.1371/journal.ppat.1014394)
Supplement: S1 File — (PDF) [file ppat.1014394.s005.pdf]

Image ID: 0000091_03
Acquire Time: Jun 17, 2025 10:38:39 PM

Image Display Values

Channel	Color	Minimum	Maximum	K
Chemi	Gray Scale (Black on White)	2.05	172	0

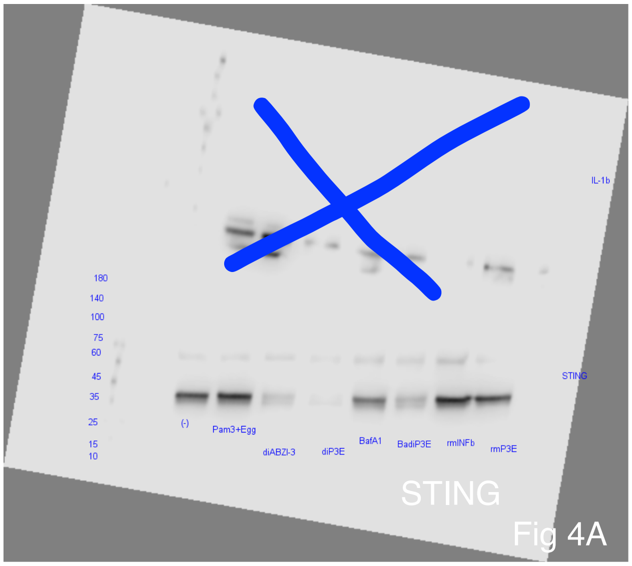

Acquisition Information

Column	Value
Image ID	0000091_03
Acquire Time	Jun 17, 2025 10:38:39 PM
Sensitivity	Standard
Image Name	0000091_03
Comment	
Image Modifications	Flip Right to Left Image ID: 0000091_01; Free Rotate 10 Image ID: 0000091_02
Project	

Image ID: 0000099_02
Acquire Time: Jun 27, 2025 12:59:26 PM

Image Display Values

Channel	Color	Minimum	Maximum	K
Chemi	Gray Scale (Black on White)	1.15	43.5	0

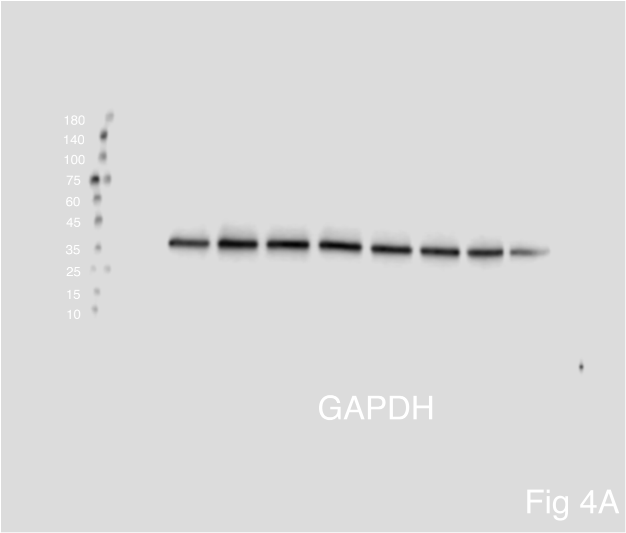

Acquisition Information

Column	Value
Image ID	0000099_02
Acquire Time	Jun 27, 2025 12:59:26 PM
Sensitivity	Standard
Image Name	0000099_02
Comment	GAPDH
Image Modifications	Flip Right to Left Image ID: 0000099_01
Project	

Image ID: 0000089_03
Acquire Time: Jun 16, 2025 3:02:59 PM

Image Display Values

Channel	Color	Minimum	Maximum	K
Chemi	Gray Scale (Black on White)	11.5	243	1

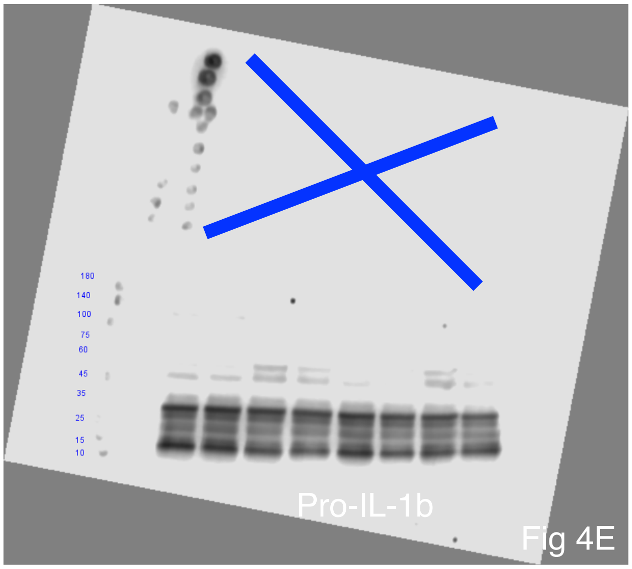

Acquisition Information

Column	Value
Image ID	0000089_03
Acquire Time	Jun 16, 2025 3:02:59 PM
Sensitivity	High
Image Name	0000089_03
Comment	
Image Modifications	Flip Right to Left Image ID: 0000089_01; Free Rotate 11 Image ID: 0000089_02
Project	

Image ID: 0000091_02
Acquire Time: Jun 17, 2025 10:38:39 PM

Image Display Values

Channel	Color	Minimum	Maximum	K
Chemi	Gray Scale (Black on White)	2.05	172	0

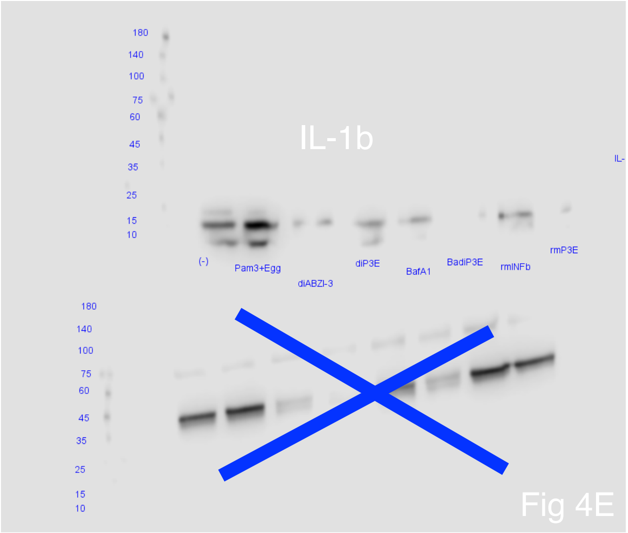

Acquisition Information

Column	Value
Image ID	0000091_02
Acquire Time	Jun 17, 2025 10:38:39 PM
Sensitivity	Standard
Image Name	0000091_02
Comment	
Image Modifications	Flip Right to Left Image ID: 0000091_01
Project	

Image ID: 0000093_03
Acquire Time: Jun 20, 2025 2:10:09 PM

Image Display Values

Channel	Color	Minimum	Maximum	K
Chemi	Gray Scale (Black on White)	2.08	124	0.5

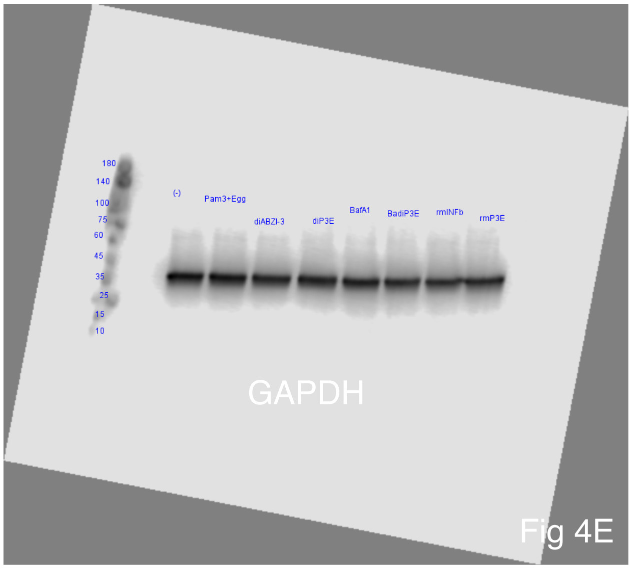

Acquisition Information

Column	Value
Image ID	0000093_03
Acquire Time	Jun 20, 2025 2:10:09 PM
Sensitivity	Standard
Image Name	0000093_03
Comment	GAPDH
Image Modifications	Flip Right to Left Image ID: 0000093_01; Free Rotate 11 Image ID: 0000093_02
Project	

Image ID: 0000024_01
Acquire Time: May 10, 2024 2:04:09 PM

Image Display Values

Channel	Color	Minimum	Maximum	K
Chemi	Gray Scale (Black on White)	2.38	80.1	0

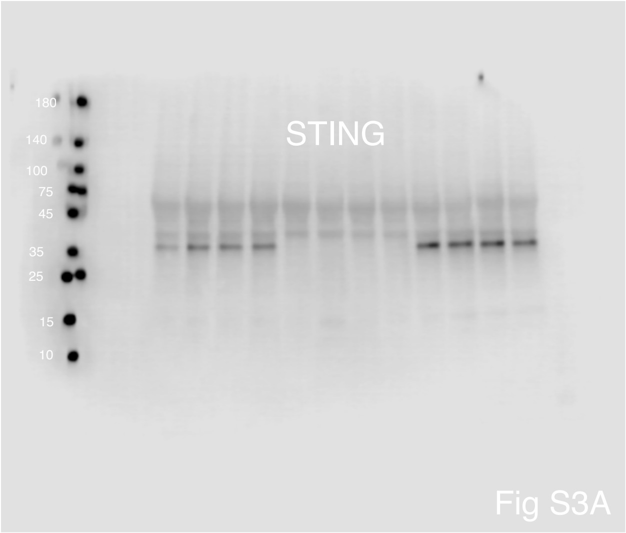

Acquisition Information

Column	Value
Image ID	0000024_01
Acquire Time	May 10, 2024 2:04:09 PM
Sensitivity	High
Image Name	0000024_01
Comment	
Image Modifications	
Project	

Image ID: 0000027_02
Acquire Time: May 11, 2024 4:43:06 PM

Image Display Values

Channel	Color	Minimum	Maximum	K
Chemi	Gray Scale (Black on White)	2.19	172	0

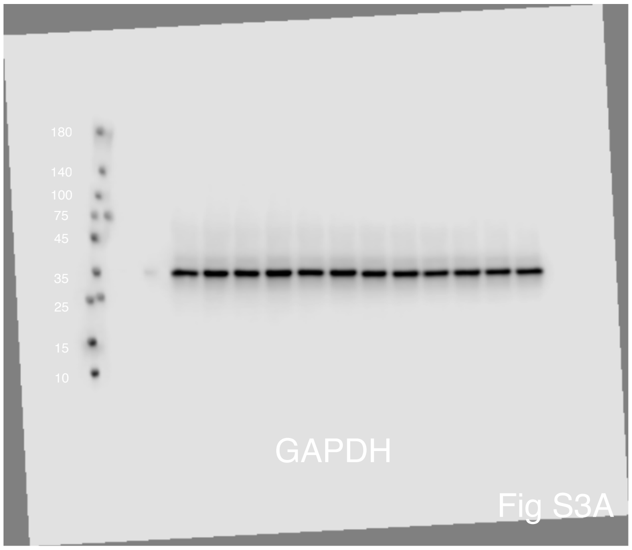

Acquisition Information

Column	Value
Image ID	0000027_02
Acquire Time	May 11, 2024 4:43:06 PM
Sensitivity	Standard
Image Name	0000027_02
Comment	gapdh #1
Image Modifications	Free Rotate 357 Image ID: 0000027_01
Project	

Image ID: 0000109_02
Acquire Time: Jul 11, 2025 3:51:50 PM

Image Display Values

Channel	Color	Minimum	Maximum	K
Chemi	Gray Scale (Black on White)	21.9	1810	0

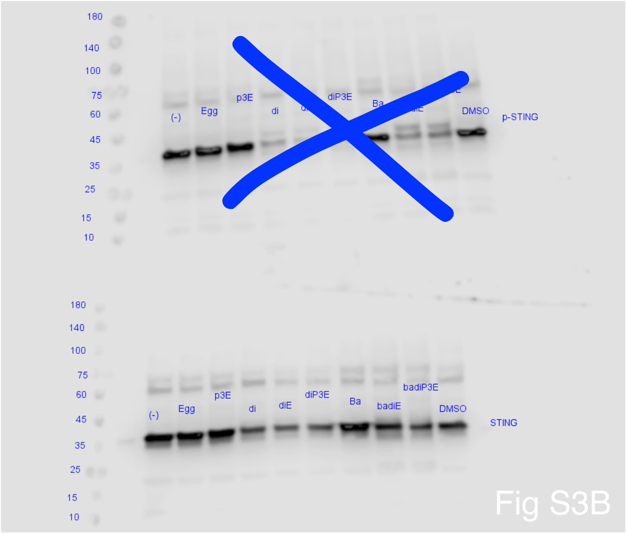

Acquisition Information

Column	Value
Image ID	0000109_02
Acquire Time	Jul 11, 2025 3:51:50 PM
Sensitivity	High
Image Name	0000109_02
Comment	1laddard:2h STING; 2laddar: 6h P-STING
Image Modifications	Flip Right to Left Image ID: 0000109_01
Project	

LI-COR®

Image ID: 0000117_02
Acquire Time: Jul 20, 2025 11:18:02 AM

Image Display Values

Channel	Color	Minimum	Maximum	K
Chemi	Gray Scale (Black on White)	23.4	2990	1

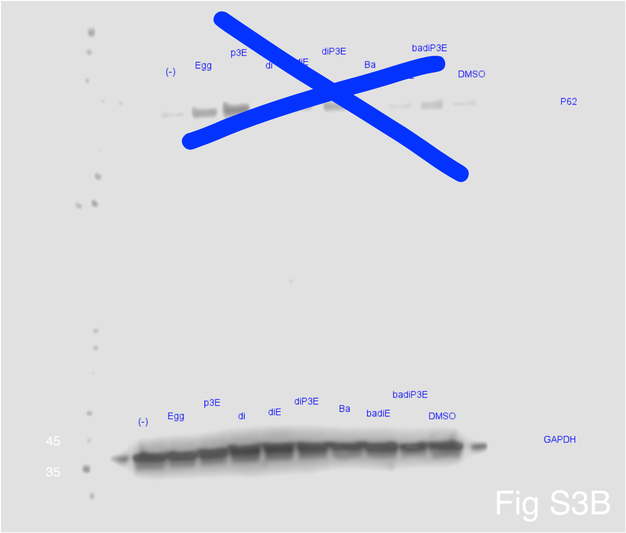

Acquisition Information

Column	Value
Image ID	0000117_02
Acquire Time	Jul 20, 2025 11:18:02 AM
Sensitivity	Standard
Image Name	0000117_02
Comment	top 6h P62 bottom 2h GAPDH
Image Modifications	Flip Right to Left Image ID: 0000117_01
Project	
